# Automated monitoring of respiratory rate as a novel humane endpoint: A refinement in mouse metastatic lung cancer models

**DOI:** 10.1371/journal.pone.0257694

**Published:** 2021-09-20

**Authors:** Caroline B. Winn, Seo-Kyoung Hwang, Jeffrey Morin, Crystal T. Bluette, Balasubramanian Manickam, Ziyue K. Jiang, Anand Giddabasappa, Chang-Ning Liu, Kristin Matthews

**Affiliations:** 1 Comparative Medicine, Pfizer Worldwide Research, Development & Medical, Cambridge, Massachusetts, United States of America; 2 Comparative Medicine, Pfizer Worldwide Research, Development & Medical, Groton, Connecticut, United States of America; 3 Global Pathology and Investigative Toxicology, Pfizer Worldwide Research, Development & Medical, Groton, Connecticut, United States of America; 4 Comparative Medicine, Pfizer Worldwide Research, Development & Medical, San Diego, California, United States of America; Southern Illinois University School of Medicine, UNITED STATES

## Abstract

In oncology research, while xenograft tumor models are easily visualized and humane endpoints can be clearly defined, metastatic tumor models are often based on more subjective clinical observations as endpoints. This study aimed at identifying objective non-invasive criteria for predicting imminent distress and mortality in metastatic lung tumor-bearing mice. BALB/c and C57BL/6 mice were inoculated with CT26 or B16F10 cells, respectively. The mice were housed in Vium smart cages to continuously monitor and stream respiratory rate and locomotion for up to 28 days until scheduled euthanasia or humane endpoint criteria were met. Body weight and body temperature were measured during the study. On days 11, 14, 17 and 28, lungs of subsets of animals were microCT imaged *in vivo* to assess lung metastasis progression and then euthanized for lung microscopic evaluations. Beginning at day 21, most tumor-bearing animals developed increased respiratory rates followed by decreased locomotion 1–2 days later, compared with the baseline values. Increases in respiratory rate did not correlate to surface tumor nodule counts or lung weight. Body weight measurement did not show significant changes from days 14–28 in either tumor-bearing or control animals. We propose that increases in respiratory rate (1.3–1.5 X) can be used to provide an objective benchmark to signal the need for increased clinical observations or euthanasia. Adoption of this novel humane endpoint criterion would allow investigators time to collect tissue samples prior to spontaneous morbidity or death and significantly reduce the distress of mice in the terminal stages of these metastatic lung tumor models.

## Introduction

Experimental animal models of metastasis are important tools for cancer research and antitumor drug discovery [1]. The choice of appropriate humane endpoints provides significant opportunities for refinement of experimental animal models [2,3]. External or heterotopic tumor models, such as those implanted on the flank, are easy to visualize and measure tumor size, volume, weight, ulceration, and/or effect on locomotion [4]. Yet, determining the tumor burden of internal orthotopic cancers or metastatic diseases, remains challenging [5]. The intentional use of death as an endpoint is highly undesirable [6]. Currently, the onset of clinical signs and confirmation of tumor presence conducted at necropsy are commonly used to monitor animal well-being and correlation to tumor progression. Alternative methods may include scheduled serial termination of animals, investigative surgery, plethysmography, and imaging techniques. Surrogate markers of tumor development such as body weight, body condition score, and body temperature may also be used to limit development of clinical signs or spontaneous animal death in this model [2]. These alternative methods can provide objective data but do have their drawbacks. Scheduled serial terminations often require higher numbers of animals and some animals may be euthanized before significant disease progression leading to a potential waste of animals. Surgical methods are invasive, requiring anesthesia and analgesia and add additional stress to an animal who may have significant disease. Imaging techniques such as real time microcomputed tomography (microCT) imaging [7–9] have been developed for monitoring lung tumor progression in mice. Although microCT imaging of mice is non-invasive, it must be performed under anesthesia, which poses additional risk in animals with metastatic lung tumors due to respiratory depression. Despite these efforts, defining humane endpoints objectively in lung metastasis mouse models remains a challenge.

Pulmonary physiological parameters such as respiratory rate are important study endpoints in phenotyping, pharmacological, safety pharmacology and toxicological studies. Plethysmography is the most common used non-invasive method of monitoring the respiratory function. Though this method does not require anesthesia, the rodent must be restrained [10], which induces stress. In addition, the small size of mice poses additional difficulties in measuring and controlling respiratory flows [11]. Cage side respiratory rate monitoring can be difficult due to the high respiratory rate of mice and only offers a snapshot of data which can be influenced by the time of day and by observer. Vium Smart Housing (Vium Digital Vivarium ™) is an emerging non-invasive video image- and cloud-based monitoring system, which continuously records and streams the respiratory rate and locomotion of a free-moving mouse in its home cage [12,13]. The aim of this study was to correlate a functional endpoint (automated respiratory rate) with subjective clinical observations and pathologic lung evaluation scores in mouse lung tumor models. We hypothesized that by using continuous monitoring with the smart caging technology we could identify pre-morbidity changes and thresholds in respiratory rate in the metastatic lung tumor models induced by the CT26 murine colon carcinoma cells and B16F10 murine melanoma cells.

## Materials and methods

### Tumor cell line culture

The CT26 murine colon cancer and B16F10 murine melanoma cell lines (ATCC, Manassas, VA, USA) were maintained in RPMI 1640 medium (Thermal Fisher Scientific, Waltham, MA, USA) containing 10% fetal bovine serum, 1% streptomycin/penicillin at 37°C in an incubator with 5% CO_2_. On day of inoculation (day 1), the cells were detached using 5% trypsin for 5 minutes and washed once with phosphate-buffered saline (PBS). The cells were then resuspended in PBS and counted using a hematocytometer.

### Animals

All housing of animals and experimental activities were carried out in AAALAC-accredited facilities in accordance with USA federal, California and Massachusetts states, local and institutional regulations and guidelines governing the use of animals and were approved by the Pfizer Institutional Animal Care and Use Committees (IACUCs).

Seven to 8-week-old female Virus Antibody Free (VAF^®^) BALB/c and C57BL/6 mice (strain codes 028 and 027, Charles River Laboratories, Wilmington, MA, USA) were used in this study after a 3-day acclimation period. The mice were group-housed in individually ventilated cages (Lab Products, Seaford, DE, USA) on enriched corncob bedding (Enrich-o’Cobs^®^, The Andersons Inc. Quakertown, PA, USA) at the Pfizer La Jolla vivarium. The rooms were temperature- and humidity-controlled (20–26°C and 30–70%, respectively) with a 12:12 h light:dark cycle. Mice were given *ad libitum* access to water (reverse osmosis and chlorinated) and standard laboratory mouse diet (5053i, LabDiet, St. Louis, MO, USA).

### Experimental procedures

Two experiments were conducted sequentially within 5 months. In both experiments, the BALB/c mice were inoculated with CT26 tumor cells, and monitored with the same set of smart cage equipment. In the second experiment, B16F10 cells were inoculated in additional subset of C57BL/6 mice.

In CT26 BALB/c experiment, 19 BALB/c mice were injected with 0.25 x 10^6^ CT26 tumor cells/100 μL/mouse intravenously (IV) via the tail vein [14], while the remaining 14 mice were injected via IV tail vein with 100 μL of PBS (controls). Mice were divided into 8 groups, 4 tumor and 4 control groups (corresponding to 4 timepoints) and singly housed in the Vium Digital Vivarium™ caging system for continuous monitoring. Body temperatures were taken prior to IV injection, approximately 2 weeks after IV injection and again prior to euthanasia. Body weights were recorded prior to injection and then again at least twice weekly. Each group was assigned a time-point for micro-CT imaging and euthanasia for lung collection, lung weight, lung photo imaging, and submission for histopathology. Animals were removed from the study early and euthanized within 14 hours when clinical observations of morbidity (e.g. hunched posture, inactivity, pale extremities, dyspnea) were noted (same for CT26 BALB/c and CT26 BALB/c with UID chip experiments). All other animals were euthanized on the last day of the experiment (study day 31).

In the second experiment, 10 group-housed BALB/c mice were injected with CT26 tumor cells and another 9 BALB/c mice were injected with PBS as controls. Additional 10 group-housed C57BL/6 mice were injected with B16F10 cells and another 9 C57Bl/6 mice were injected with PBS. Different from animals in CT26 BALB/c experiment, all mice were implanted with UID microchips (UC-1485, UID Identification Solutions, Lake Villa, Illinois, USA) in the right flank prior to shipment from the vendor. UID temperature microchip (UCT-2112) were implanted in the left flank in house for body temperature monitoring. Body temperatures were taken prior to IV injection, approximately 2 weeks after IV injection and prior to euthanasia. All animals were euthanized before or on study day 33.

### Vium digital vivarium^®^ technology

On day 4 (CT26 BALB/c with UID chip and B16F10 C57BL/6 with UID chip experiments) or 7 (CT26 BALB/c experiment) post-injection, the animals were shipped to the Pfizer vivarium in Cambridge, MA, USA. The animals were singly housed in Innovive individually ventilated cages with diced cellulose bedding (ALPHA-dri^®^, Shepherd Specialty Papers Inc., Watertown, TN, USA) and nesting material (Nestlet, Ancare Corp., Bellmore, NY, USA) in Vium (Vium, Inc., San Mateo, CA, USA) racks with artificial 12h light:12h dark cycle (3500 K LED) fed standard chow pellets (5053i, LabDiet, St. Louis, MO, USA) and chlorinated (1.0–3.0 ppm) reverse osmosis water via pre-filled water bottles *ad libitum* (Aquavive^®^, Innovive, San Diego, CA, USA). Animals were housed in temperature- and humidity-controlled rooms (20–26°C and 30–70%, respectively). Experimental methods using Vium smart cages were reported in our previous mouse study [15]. Briefly, Vium Digital Smart Cages were outfitted with cameras and sensors that stream animal data 24 hours a day, 7 days a week to a cloud-based data infrastructure. The Vium Digital Platform obtained and displayed the following information continuously in near real-time: (1) conducted procedures with corresponding times; (2) data analytics on motion and respiratory rate; and (3) verification of illumination. This study used the validated Vium Motion metric and Vium Breathing Rate metric only. Breathing rate data (average of 1 hour) greater than 250 breath/min (BPM) were filtered during day 1 to day 17.

### Clinical observations

Animals were inspected at least once daily from day 1 to day 12 and then twice daily from day 13 to experimental endpoint. Assessments were performed by a clinical veterinarian, and/or trained veterinary or laboratory animal technicians. Animal observations ranged from mild, moderate to severe in grades of respiratory rate and effort. Locomotion, postural changes and behavior were also noted. In CT26 BALB/c experiment, body temperature was obtained before scheduled euthanasia and non-scheduled euthanasia (at time of meeting humane endpoints) with a non-contact digital infrared thermometer (Lasergrip 774, Etekcity Inc. Anaheim, CA, USA) by gently restraining the animals by the scruff and laser pointed approximately 5 cm from the sternum. If an animal was flagged for disease-related clinical assessment, body weight was recorded daily until the fate of the mouse was determined. In CT26 BALB/c with UID chip and B16F10 C57BL/6 with UID chip experiments, body temperatures were measured with a UID reader once or twice a day.

### MicroCT imaging

MicroCT (SkyScan 1276 microCT system, Bruker, Kontich, Belgium) scanning procedures were adopted from our previous work [16] for CT26 BALB/c experiment only. Briefly, the following parameters were used: voxel resolution 84 μm, tube setting 55 kV, 200 μA, with a rotation step of 0.7°, 62 ms exposure time and in 360° scan mode. Flat fields were corrected. Beam hardening reduction was applied using a 0.5 mm Al filter. The total scanning time for each animal (the thorax) was approximately 6–7 min without averaging. The camera was set to low resolution (336 x 504), 8 binning. The mice were positioned in supine and facing the operator on an animal bed (diameter 30 mm) with real-time visual monitoring of the animal position, respiratory rate and chamber temperature. All microCT image data were acquired in free-breathing mice under isoflurane anesthesia (1.5–2.5%) without respiratory or cardiac gating. The temperature of the animal bed chamber was maintained at 28–30°C to prevent hypothermia during scanning. The projection images were reconstructed to TIF file type for subsequent analysis. The following correction was applied: smoothing = 1; ring artifact = 1; beam hardening correction = 12; histogram cut offs: 0.001–0.03.

### Necropsy and histopathology

Animals were euthanized by CO_2_ asphyxiation. The lungs were collected, macroscopically evaluated and weighed (CT26 BALB/c experiment only) following confirmation of death, and inflated and fixed in 10% neutral buffered formalin (NBF) [17]. Dorsal and ventral pictures of the lung were captured for illustration of macroscopic nodules [18]. Representative samples of lung tissues were fixed in 10% neutral buffered formalin, embedded in paraffin, sectioned at 5 μm, and stained with hematoxylin and eosin (H&E). All lung tissues collected were examined microscopically from all animals by a Veterinary Anatomic Pathologist. The pathology scores were derived from representative sections of lungs (including the bronchus and the pleura). Microscopic tumor load in the lungs were measured and graded on a scale of 0 to 100% based on the percentage of lung section affected by the neoplasm to evaluate tumor metastasis.

### Statistical analysis

All the results are reported as mean ± standard deviation (SD) or standard error of the mean (SEM). The data were statistically analyzed by Student’s *t*-test or two-way analysis of variance (ANOVA) followed by Dunnett’s or Bonferroni’s post hoc test using Graphpad Prism 8 software (La Jolla, CA, USA). P values of less than 0.05 (*) were considered statistically significant.

## Results

### Clinical observation and lung metastasis in CT26 BALB/c mouse model

**Fig 1:**
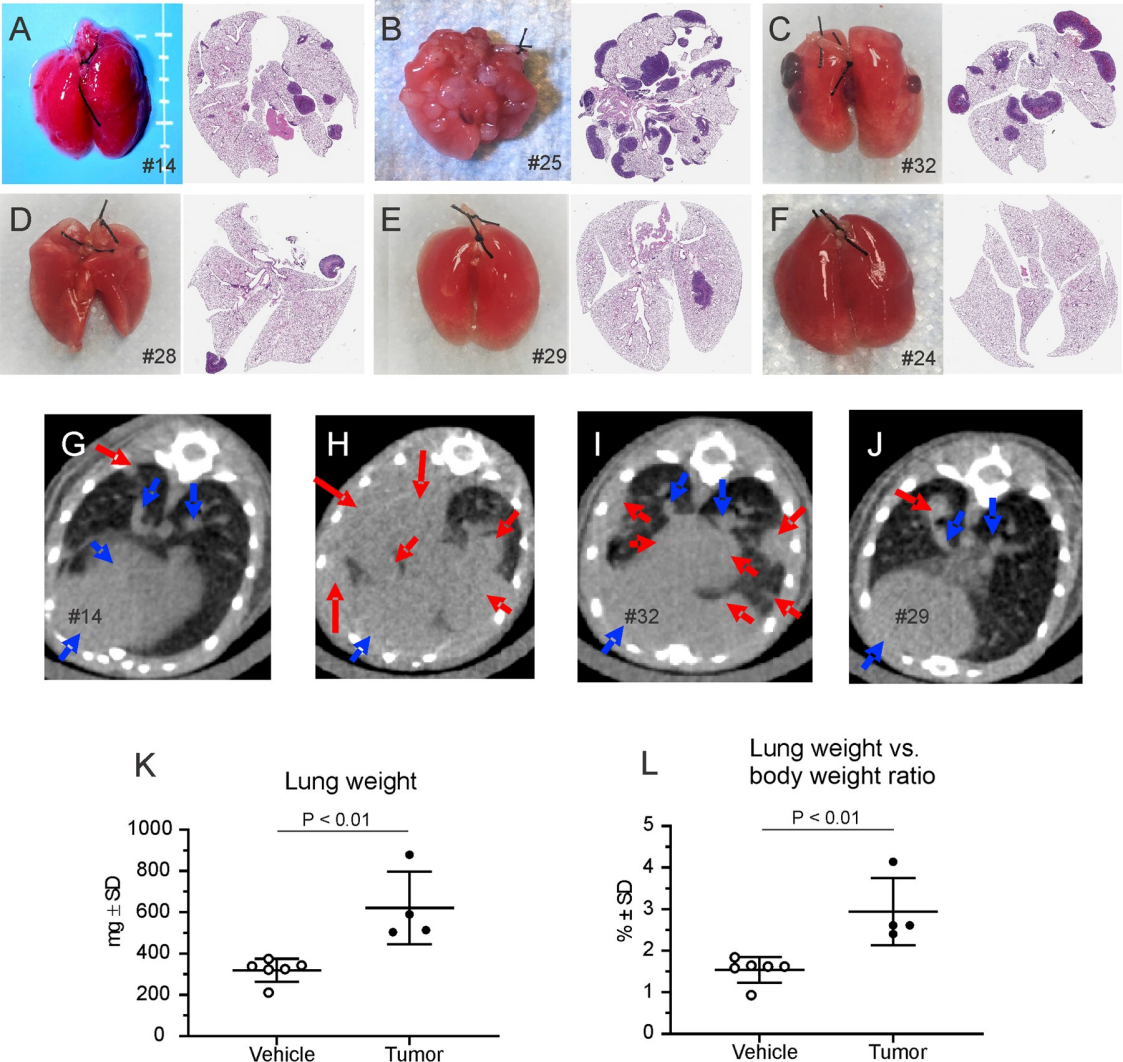
Representative photos and micrographs of CT26 tumor-bearing lungs (A-E), normal lungs (F), lung weights (K), lung weight vs body weight ratio (L), and microCT imaging of lungs (G-J). The majority of CT26 cell-injected animals developed metastases in their lungs with multiple nodules on the surface of the lungs (A-D). The metastatic lungs had statistically significant increased weights (K, P < 0.01, unpaired t-test) and lung weight-body weight ratio (L, P < 0.01, unpaired t-test), compared with that of the vehicle injected animals. On the microCT images, blue arrows indicate normal tissue and red ones indicate abnormal tissue (tumor).

The first sign of illness was ruffled fur on day 24 after CT26 cell injection in CT26 BALB/c experiment and day 19 in CT26 BALB/c with UID chip experiment. Visible signs of illness peaked on days 27–28 (CT26 BALB/c experiment) or 21–23 (CT26 BALB/c with UID chip experiment) and consisted of ruffled fur, reduced activity, and pale-colored pinnae (n = 3 for CT26 BALB/c experiment and n = 8 for CT26 BALB/c with UID chip experiment). Clinical observations for animals with and without UID chips implanted on days of increased breathing rate were noted–respiratory signs ranged from mild to severe for tumor-bearing animals (Table A in S1 File). Table 1 presents the lung tumor metastasis outcome of the animals which received CT26 cell injection (n = 19 in CT26 BALB/c experiment and n = 10 in CT26 BALB/c with UID chip experiments). In CT26 BALB/c experiment, on day 11, no lung tumors were observed in any of the animals (0/4). Photographs and histology images show the typical metastatic lung tumors in CT26 model (Fig 1A–1F). On day 14, only 1 out of 4 animals (#14) had multiple small nodules on the surface of the lung (Fig 1A, left) and minimal changes on microCT image (Fig 1G). On days 24–28, 6 out of 13 animals had overt metastatic tumors in the lungs with multiple circumscribed pale white nodules (Fig 1B), severe pulmonary changes on microCT image (Fig 1H), increased lung weights (Fig 1K) and lung weight/body weight ratio (Fig 1L). Retrospective respiratory rate analysis was performed only in these 6 animals (Table 1). One animal (#32) with a few dark red-colored nodules (similar in appearance to hemangioma) on the surface of the lung (Fig 1C) had microCT evidence of solid tumors within the lung parenchyma (Fig 1I). MicroCT imaging (Fig 1J) also revealed abnormal tissue in the lungs of animals with only two (#28, Fig 1D) or no (#29, Fig 1E) nodules visible on the surface of the lung. Two tumor-bearing animals were found dead before meeting criteria for euthanasia due to lung metastasis. In CT26 BALB/c with UID chip experiment, 8 animals had overt metastatic tumors in the lungs with multiple circumscribed pale white nodules. The other 2 animals (#24 and #30) had only a few flat white spots on the surface of lungs (images not shown). Three tumor-bearing animals were found dead before meeting criteria for euthanasia due to lung metastasis.

**Table 1:** Time courses of breathing rate and motion speed in animals with overt lung metastasis.

Experiment (Tumor type) (Animal strain)	Animal Number	Day when breathing rate began to increase	Day when motion speed began to decrease	Fold changes in the breathing rate when motion speed decreased (average of 11AM-12 PM)	Days between breath rate increase and motion decrease	Estimated Tumor nodule count (n)	Lung weight (mg)	Lung pathology score (%)	Clinical Observation on day when breathing rate began to increase
CT26 BALB/c	25	27	29	1.5*	2	60	589.2	50	Mild respiratory rate (RR) & effort (RE), active, well-groomed coat
	26	24	27	1.7	3	45	514.1	50	Mild respiratory signs, active, nesting, well-groomed coat
	27	26	27	1.4	1	61	878.6	75	Moderate respiratory signs, nesting, well-groomed coat
	30	26	27	1.7	1	77	504.0	50	Severe respiratory signs, hunched, pale pinna, low activity
	31	23	24	2.1	1	79	n/a	80	Moderate respiratory signs, active, well-groomed coat
	33	25	27	1.5	2	82	n/a	80	Mild respiratory signs, nesting, active, well-groomed coat
	Mean ± SD	25.2 ± 1.3	26.8 ± 1.5	1.7 ± 0.2	1.7 ± 0.7	67.3 ± 13.2	621.5 ± 152.1	64.2	
CT26 BALB/c with UID chip	22	21	21	1.6*	0	89	n/a	50	Moderate-severe RR/RE, ears pinned, eyes squinted, mild piloerection
	27	20	21	1.8	1	69	n/a	40	Moderate RR/RE, Slight hunch, piloerect, eyes squinted, ears pinned
	28	19	21	2.0	1	115	n/a	40	Severe RR/RE, eyes squinted, ears pinned, piloerect, hunched
	33	20	21	1.9	1	85	n/a	40	Mild RR/RE, hunched, piloerect, ears pinned, eyes squinted
	34	20	22	1.6	2	86	n/a	50	Moderate RR/RE, hunched, piloerect, ears pinned, eyes squinted
	36	19	20	1.7	2	125	n/a	40	Severe RR/RE, piloerect, hunched, ears pinned, eyes squinted
	38	20	21	1.4	1	101	n/a	40	Severe RR/RE, Pale, eyes squinted, ears pinned back, hunched, severe piloerection
	40	22	22	1.6	0	73	n/a	30	Severe RR/RE, hunch, pale. Ears pinned; eyes squinted
	Mean ± SD	20.1 ± 0.9	21.0 ± 0.7	1.7 ± 0.2	1.0 ± 0.7	92.9 ± 18.3	n/a	41.3	
B16F10 C57Bl/6 with UID chip	64	23	23	1.2*	0	67	n/a	40	Normal, tail bruising
	66	22	23	1.1	1	66	n/a	40	Hunched, ears pinned, eyes squinted
	68	20	23	1.3	2	66	n/a	30	Mild RR/RE, piloerect
	70	26	29	1.6	3	99	n/a	60	Moderate RR/RE, scruffy coat
	74	22	24	1.3	2	63	n/a	50	Normal
	76	20	23	1.7	3	64	n/a	60	Slight piloerection
	78	25	N/A	N/A	N/A	49	n/a	40	Mild RR/RE
	80	24	28	1.5	4	53	n/a	40	Mild RR/RE
	Mean ± SD	22.8 ±2.0	24.7 ± 2.4	1.4 ± 0.2	2.1 ± 1.2	65.9 ± 14.0	n/a	45.0	

*: Compared with the average breathing rate on day 16.

### Increase in breathing rate preceded a decrease in motion speed in BALB/c mice with CT26 cells

**Fig 2:**
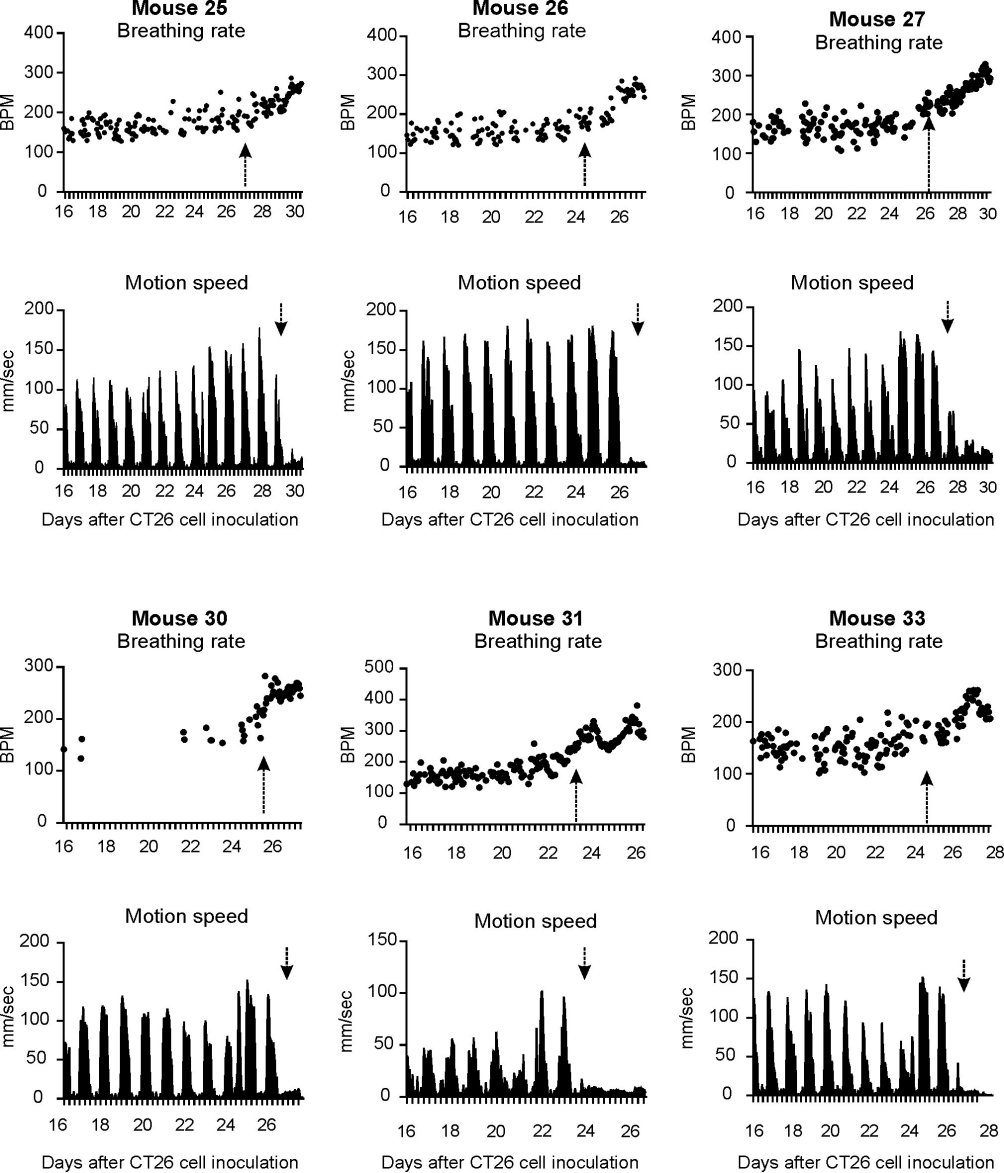
Breathing rate and motion speed changes in 6 CT26 tumor-bearing mice from day 14 to 28 in CT26 BALB/c experiment. Arrows indicate when breathing rates increased and nighttime motion speed decreased or disappeared in these animals. Each data point represents the average of the breathing rates collected over 1 hour’s duration. Nighttime motion speeds were much greater than the daytime values. X-axis division markers represent 6 hr intervals. Note that the respiratory movements were not detected at sometimes between days 14–22. This was due to the poor contrast of colors of bedding with animals’ white coat. The issue was resolved after bedding changes.

**Fig 3:**
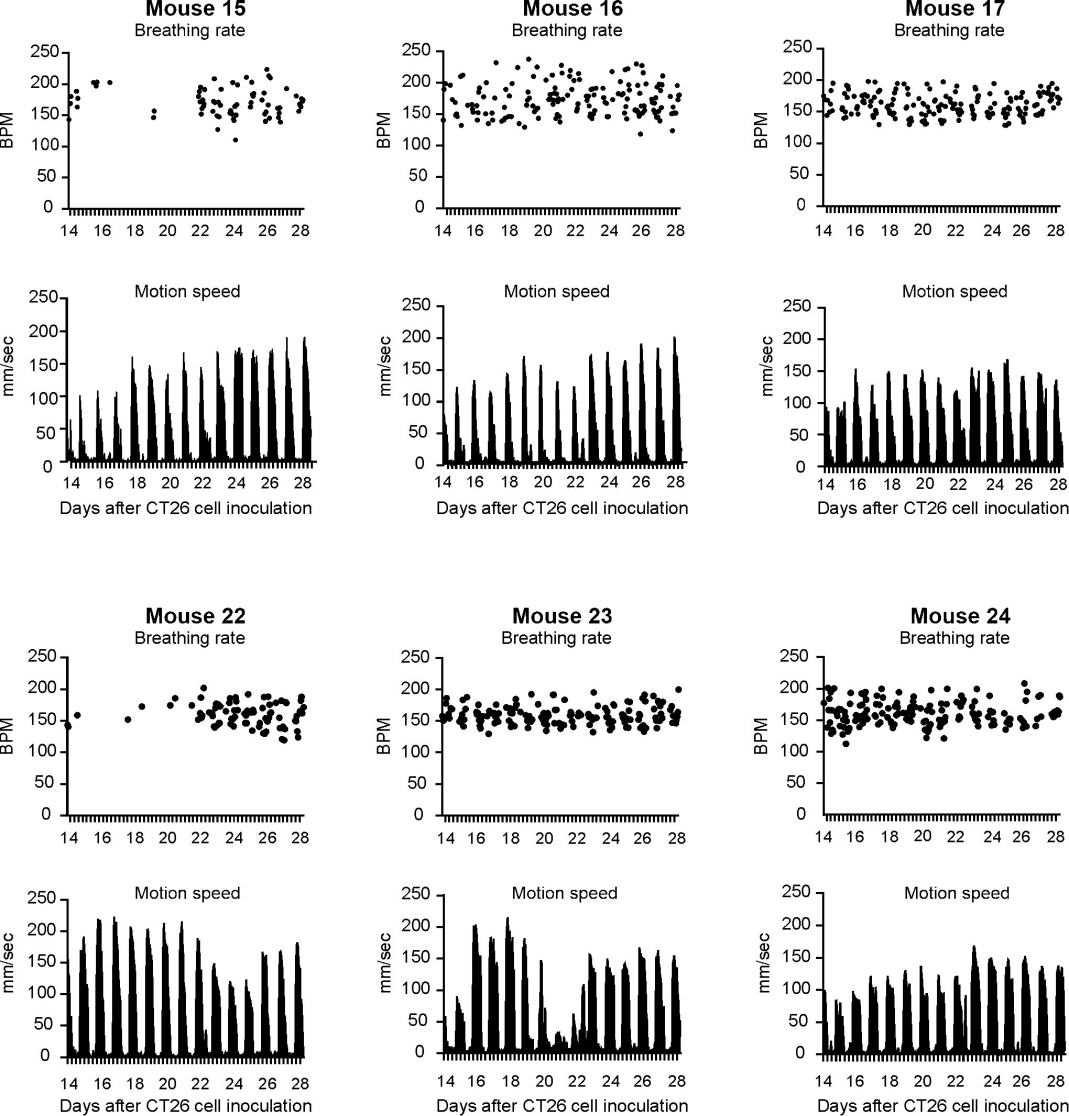
Breathing rate and motion speed in 6 vehicle injected BALB/c mice from day 14 to 28 in CT26 BALB/c experiment. There were no changes in breathing rate or motion speed during the 14 days.

**Fig 4:**
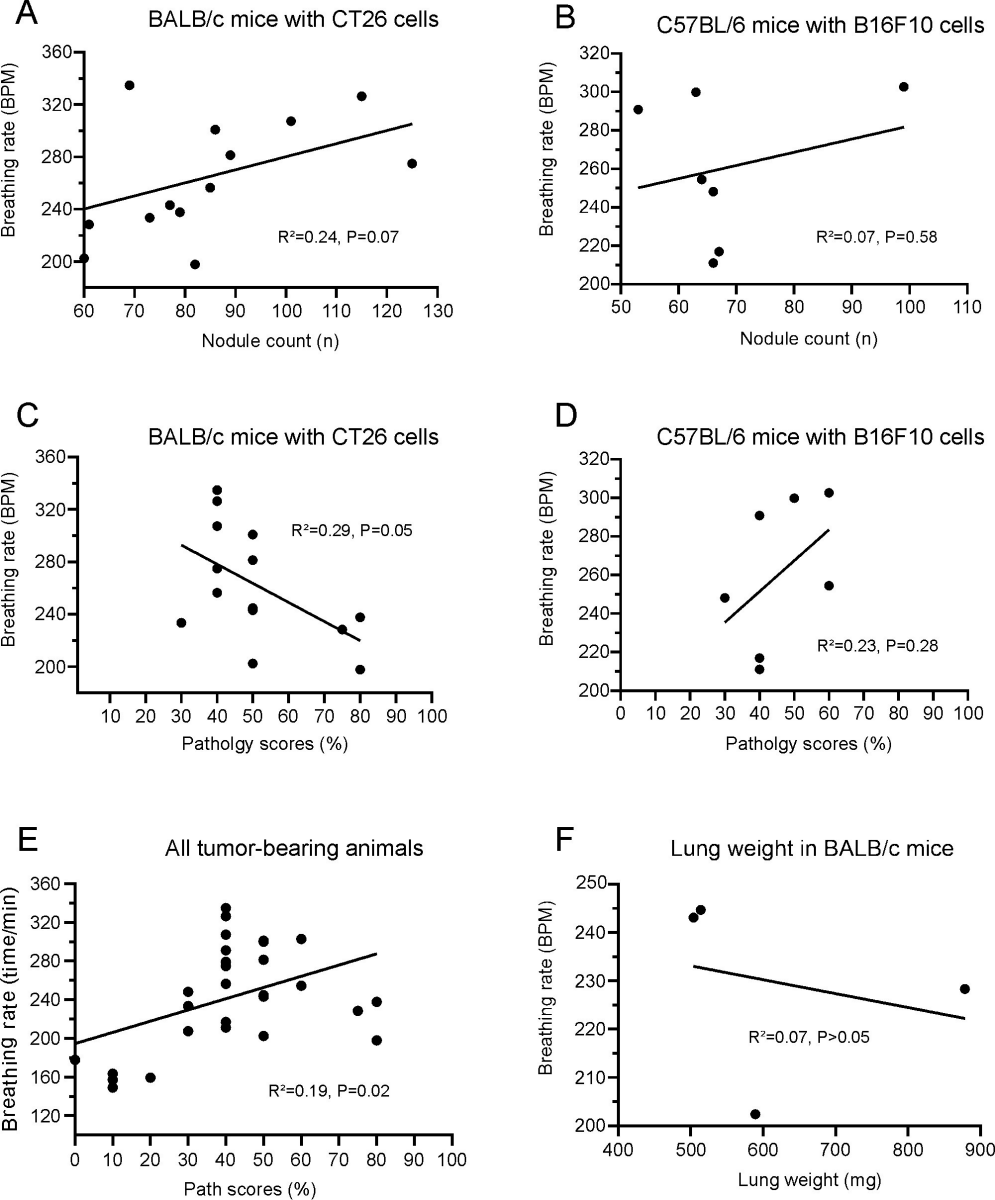
No correlations of increased breathing rates to lung tumor nodule counts, pathology score and weight in BALB/c and C57BL/6 mice. The increased breathing rates were not correlated to either lung surface tumor nodular counts (A&B), pathology scores (>30%, C&D) or lung weights (F, all P > or = 0.05). However, when the data from tumor-bearing animals with pathology score < 30% were included, the breathing rate and pathology score were significantly correlated (E).

**Fig 5:**
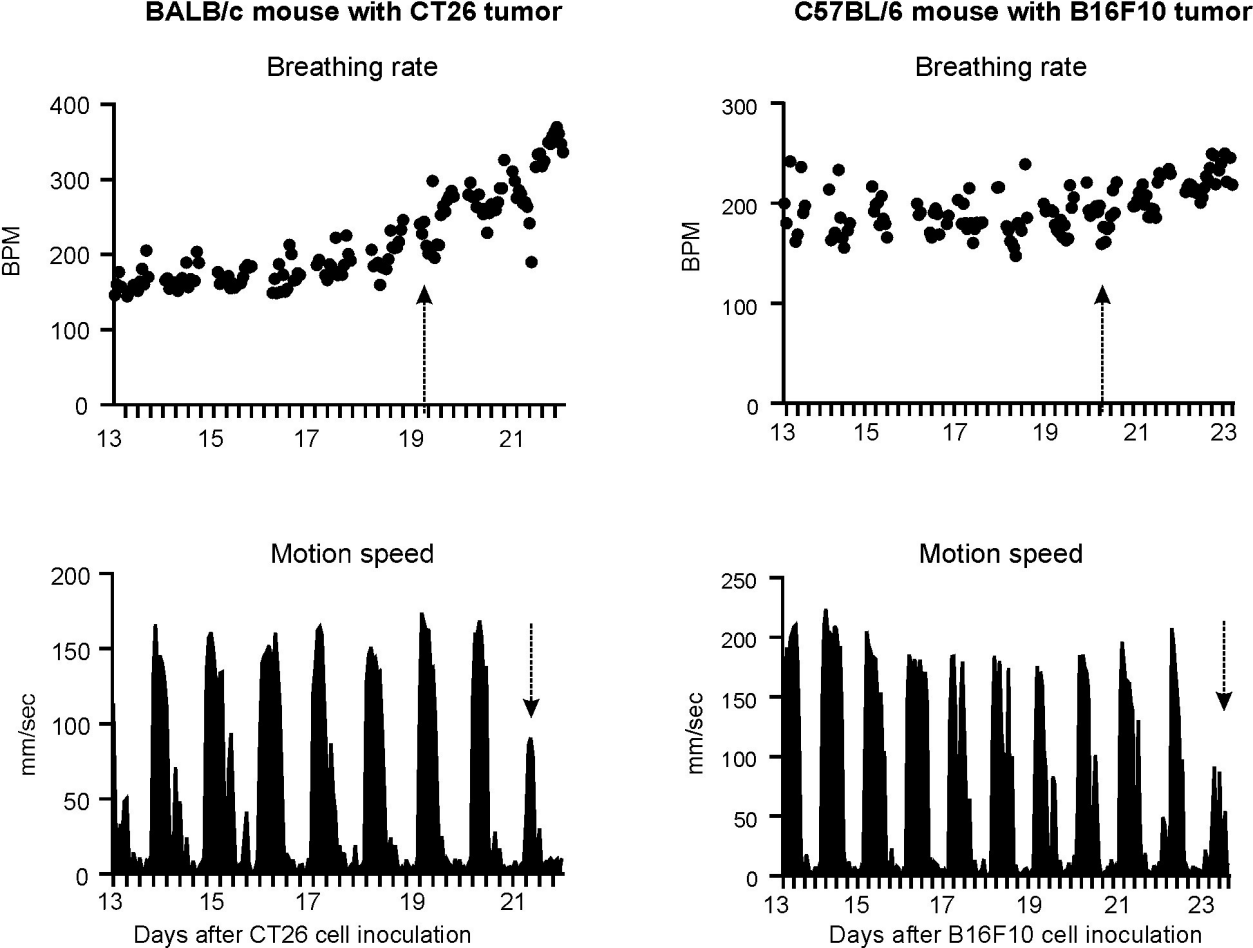
Representative charts of increased breathing rates and reduced motion speed in a BALB/c mouse bearing CT26 tumors (A) and a C57BL/6 mouse bearing B16F10 tumors (B) in CT26 BALB/c with UID chip and B16F10 C57BL/6 with UID chip experiments. Arrows indicate when breathing rates increased and nighttime motion speed decreased in these animals. Each data point represents the average of the breathing rates collected over 1 hour’s duration. X-axis division markers represent 6-hour intervals.

In CT26 BALB/c experiment, 6 out of 9 BALB/c mice with metastatic tumors in the lungs began to manifest increased respiratory rates from days 23–26 (25.2 ± 1.3) accompanied with decreased locomotion speed from days 24–29 (26.8 ± 1.5), compared with the baseline values. Table 1 and Fig 2 show the initial days of breathing rate increases, motion speed decreases and the breathing rate values when the motion speed began to decrease. When the animals began to show decreased motion speed, the corresponding average breathing rate was 246.4 ± 28.0 BPM, which is significantly higher than that of the other 6 vehicle mice from days 23–29 (163.5 ± 6.3 BPM, P < 0.001, unpaired *t*-test, Table A in S1 File). In contrast, the vehicle injected animals did not show any changes in breathing rate between days 14 to 28 (Fig 3 and S2 File). Interestingly, detailed analysis indicated that the breathing rate increase did not correlate to either the estimated counts of tumor nodules (R^2^ = 0.24, P > 0.05, Fig 4A), lung pathology score (R^2^ = 0.29, Fig 4C) or lung weight (R^2^ = 0.07, Fig 4F). In CT26 BALB/c with UID chip experiment, 8 out of 10 animals with metastatic tumors in the lungs began to manifest increased respiratory rates from days 19–22 (20.1 ± 0.9) accompanied with decreased motion speed from days 20–22 (21.0 ± 0.7), compared with the baseline. Table 1 and Fig 5 and S6 and S12 Files show the initial days of breathing rate increases, motion speed decreases and the breathing rate values when the motion speed begins to decrease. When the animals began to show decreased motion speed, the corresponding average breathing rate was 290.7 ± 37.6 BPM, which is statistically significantly higher than that of other 8 control mice from days 17–22 (170.6 ± 10.7, P < 0.001, unpaired *t*-test, Table A in S1 File). In contrast, the control animals did not show breathing rate changes from days 13 to 22 (Fig A in S1 File). Further analysis indicated that the breathing rate increase did not correlate to either the estimated counts of tumor nodules (R^2^ = 0.24, P > 0.05, combined with the data from CT26 BALB/c experiment, Fig 4A). The lung pathology scores of the tumor-bearing animals showing increased breathing rate ranged from 30–80%, while those without breathing rate increases had pathology scores ranged from 10–40% (Table 1 and Table B in S1 File).

### No changes in body weight or temperature in BALB/c mice with CT26 cells

The CT26 tumor-bearing and vehicle-injected animals showed similar body weight gain from days 14 to 28 (CT26 BALB/c experiment) or days 13–22 (CT26 BALB/c with UID chip experiment), throughout which breathing rates increased followed by motion speed decreases. There were no statistically significant differences in body weights between the tumor-bearing and vehicle animals in both experiments (all P > 0.05, two-way ANOVA, Fig B in S1 File). In CT26 BALB/c experiment, body temperatures prior to scheduled euthanasia for tumor-bearing animals ranged from 33.5 to 35.3˚C (mean ± SD: 34.3 ± 0.5˚C, n = 6). These were not significantly different from temperatures retrieved at day 14 (P > 0.05, *t*-test). A decrease in body temperature was observed in two tumor-bearing mice prior to euthanasia: 27.2˚C from 34.4˚C for animal number 27 and 29.7˚C from 33.9˚C for animal number 31. In CT26 BALB/c with UID chip experiment, CT26 tumor-bearing and vehicle-injected animals showed similar temperatures from days 7 to 25. However, when temperature data from both experiments were pooled, there were no statistically significant differences in body temperatures taken in morning (Fig C in S1 File) or in afternoon throughout the study courses (Fig C in S1 File, all P > 0.05, mixed-effect analysis).

### Clinical observation and lung metastasis in C57BL/6 mice with B16F10 cells

The first sign of illness was ruffled fur on day 24 after B16F10 cell injection. Visible signs of illness peaked on days 27–28 and consisted of ruffled fur, reduced activity, and pale-colored pinnae (n = 2). Clinical observations for animals on days of increased breathing rate were noted–respiratory signs ranged from mild to severe for tumor-bearing animals (Table A in S1 File). Table 1 presents the lung tumor metastasis outcome of the animals which received B16F10 cell injection (n = 10 in B16F10 C57BL/6 with UID chip experiment). Eight animals had overt metastatic tumors in the lungs with multiple circumscribed black nodules. The other 2 animals (#62 and #72) had only a few nodules on the surface of lungs. Three tumor-bearing animals were found dead before meeting criteria for euthanasia due to lung metastasis.

### Increase in breathing rate preceded a decrease in motion speed in C57BL/6 mice with B16F10 cells

Out of 10 animals, 8 mice with metastatic lung tumors began to manifest increased respiratory rates from days 20–26 (22.8 ± 2.0), and 7 of the 8 animals showed decreased motion speed from days 22–29 (24.7 ± 2.4), compared with the baseline. Table 1 shows the initial days of breathing rate increases, motion speed decreases and the breathing rate values when the motion speed begins to decrease. When the animals began to show decreased motion speed, the corresponding average breathing rate was 257.0 ± 46.1 BPM, which is statistically significantly higher than that of other 8 vehicle mice from days 20–28 (200.1 ± 9.7 BPM, P < 0.001, unpaired *t*-test, Table A in S1 File). In contrast, the control animals did not show breathing rate changes from days 13–30 (Fig D in S1 File). Further analysis indicated that the breathing rate increase did not correlate to the estimated counts of tumor nodules (R^2^ = 0.07, P > 0.05, Fig 4B). The lung pathology scores of the tumor-bearing animals showing increased breathing rates ranged from 30–60%, while those animals without breathing rate increased had pathology scores ranged from 0–30% and did not manifest an increase in breathing rate (Table 1 and Table B in S1 File).

### No body weight or temperature changes in C57BL/6 mice with B16F10 cells

The B16F10 tumor-bearing and control animals showed comparable body weight gain (P > 0.05, from days 14 to 26, Fig B in S1 File) and body temperature (P > 0.05, from days 6 to 26, Fig 3). There were no statistically significant differences in body temperatures taken in morning or in afternoon (Fig C in S1 File, all P > 0.05, mixed-effect analysis).

## Discussion

The choice of appropriate humane endpoints provides a significant opportunity for refinement of experiments using animals. Studies can often be terminated as soon as progressive tumor growth is evident [19]. However, in the metastatic lung tumor model, imaging techniques are currently the only available non-invasive way to directly measure tumor growth. Humane endpoints in models of lung disease are often based on subjective observations, for example, labored breathing, increased respiratory rate, decreased activity, hunched posture and ruffled fur. Measurements of these can be highly variable depending on the individual performing the observation and/or the time of day performed. Dyspnea and tachypnea are commonly reported clinical signs associated with lung disease; however, monitoring of these signs in rodents can be quite challenging due to the high basal respiratory rate (80–230 bpm, see Table A in S1 File).

Currently, whole-body plethysmography is considered the gold standard for measurement of lung function, including respiratory rate, in laboratory rodents [10]. Increases in breathing rate have been previously detected in mice with pneumonitis and lung fibrosis [20–22]. Although mice are usually acclimatized to the measurement environment, any restraint that is used remains stressful to the animals and the results obtained represent only cross-sectional data. Vium’s Digital Vivarium^™^ is a developing technology in which sensors and high-definition cameras, coupled with computer vision, data algorithms, and cloud capabilities allow for continuous monitoring of animals and collection of automated metrics such as motion speed and breathing rate. This technology has been used successfully in monitoring lipopolysaccharide (LPS)-induced breathing rate increases in mice accompanied by decreased motion speed as early as 1 hour after LPS administration [23]. In our study, the normal breathing rate of control animals and early time of tumor-bearing animals is lower than previously reported (Table C in S1 File) but within the published range of 80–230 BPM in healthy, unstimulated mice [24]. The difference between our measured baseline breathing rate and values reported in the literature may be a result of the testing conditions as animals are often restrained during plethysmography testing, in addition to user technique, sex, genetic regulation, and environmental variables, such as temperature. Due to these differences, we advocate capturing baseline assessments prior to disease progression, as they likely are specific to rodent strain, environment, and disease phenotype.

Most (72%) of the CT26 or B16F10 tumor-bearing animals developed an increased respiratory rate by days 21–26 followed by decreased motion speed in 1–2 days. Respiratory rates increased approximately 1.4 folds, from a baseline of 164 ± 6 breaths per minute (BPM) to 246 ± 28 BPM (CT26 model). Tumor nodule counts and lung weights were not correlative with increased respiratory rates and it is likely that once a certain threshold of lung volume is compromised, that respiratory rate will be elevated, which would not show a linear trend. In addition, estimated surface count of tumor nodules may not be representative of tumor burden of the entire organ. This study demonstrates that automatically monitored breathing rates captured with Vium technology can be used to establish an earlier humane endpoint in a mouse model of metastatic lung cancer. The observed increases in breathing rates preceded locomotion decreases or clinical signs of morbidity by 1–2 days in animals with severe tumor metastases in the lungs in both models, some of which were confirmed with microCT and necropsy. Interestingly, in both models, animals showing increased breathing rates had lung pathology scores above 30%. The tumor-bearing animals with pathology scores below 30% did not exhibit increased breathing rate during the observed course up to day 28. Further analysis revealed that while all data from tumor-bearing animals were pooled, the breathing rate and pathology score are correlated (Fig 4E). However, breathing rate increases beyond the threshold of 30–40% did not correlate to pathology scores in either model (Fig 4C and 4D). Additionally, we noted that the average onset of increased breathing rate in CT26 tumor-bearing mice was slightly different between those without chip and with chip implanted (day 25.2 vs. 20.1). It is unlikely that chip implantation alone caused this change, since a previous study found no significant impact of microchipping in phenotyping test performance, including locomotion, auditory and visual morphology and function, and general health and welfare in mice [25]. Rather, the difference in onset might be an intrinsic feature of the CT26 BALB/c model. For instance, in a previous study [18], about 60% animals died between day 20–30, due to severe pulmonary metastasis. In vivo imaging, such as microCT [9] or bioluminescent imaging, could provide more accurate and precise information about tumor burden. However, the inflexibility in imaging scheduling prevented the application, especially at the late stages. In addition, in our experiments with UID temperature implanted, the unexpected chip-generated artifacts seriously impact the image analysis. Clinical observations on day of increased breathing rates detected by the system ranged from mild to severe for tumor-bearing animals. Thus, while clinical signs are helpful to flag animals for increased monitoring, they are not as sensitive as captured breathing rate changes. Thus, we recommend using both subjective clinical observations and objective, digitally captured breathing and locomotion rates as complementary tools to guide humane endpoint decisions. Although body weight loss was reported as a marker of disease progression in murine lung tumor models [26,27], we showed no changes in body weight decreases in theses lung metastasis models, similar to the results reported by Weng et al. [18]. This is also in agreement with studies reporting that the body weights did not decrease up to 48 days in BALB/c mice bearing CT26 cells [18] and no body weight decrease in B57BL/6 mice bearing B16F10 cells [28]. Hypothermia is reportedly a common sign of the moribund condition in mice. Only two mice registered decreases in body temperature prior to euthanasia, suggesting that hypothermia is not a reliable humane endpoint in these models. Therefore, the use of microchips with an integrated temperature biosensor to collect point-in-time body temperature is not required. A study evaluating remote, continuous body temperature monitoring using microchips with temperature biosensors providing additional body temperature data is needed at the time of clinical decline to assess reliability of regular temperature assessments as a measure to aid humane endpoint review–a contributing variable could have been restraint for collection for mice that had their temperature taken manually which may temporarily increase body temperature.

Anecdotally, the system also afforded timely clinical observations that otherwise may have gone unnoticed or left circumstances unclear: two mice were found dead behind the running wheel; at the time it was uncertain if they had gotten stuck. Upon review of the video feed, it was clear that the mice were in terminal condition with agonal breaths and hid behind the wheel, enabling us to conclude that the wheel was not a danger to animal welfare. Another mouse with its tail accidently caught between the cage lid and water bottle holder was caught by low activity alert. Access to the video allowed us to identify the condition readily and correct faster than by scheduled in-person checks. This type of monitoring provides another notable advantage: the ability to receive automatic alerts once the breathing rate has increased compared with previous days’ data. Thus, this technology allows investigators more time for preparation of euthanasia and tissue collection in addition to preventing severe clinical morbidity and spontaneous mortality. Remote monitoring of these parameters in near real-time also facilitates global collaboration. Potential application of this humane endpoint refinement and the smart caging technology in other lung disease models, especially primary tumor models, warrants further investigation. While the Vium smart vivarium system is an emerging technology with added costs, like most novel technologies, with increased use and competition, it is most likely that these associated costs will decline over time. Though this system at time of study was only able to accommodate single mouse per cage, multi-rodent housing has been under development [29], further reducing associated per diem costs and promoting animal welfare. As well, when the objective endpoints in these continuous monitoring systems are found to be more reliable and defined across different murine models, this may reduce the need for additional animal numbers, further promoting the reduction in use of animals per study overall.

## Conclusions

In both CT26 and B16F10 mouse metastatic lung tumor models, the respiratory rate was increased prior to motion decrease and other endpoints. We propose that increases in respiratory rate (1.3–1.5 X) can be used to provide an objective benchmark to signal the need for more frequent clinical observations or euthanasia. Adoption of this novel humane endpoint criterion would allow investigators time to collect terminal tissue samples prior to spontaneous morbidity or death and significantly reduce the distress of mice in the terminal stages of such metastatic lung tumor models.

## Supporting information

S1 File Additional tables and figures. (DOCX)

S2 File Breath data for Fig 3. Balbc control. (PZFX)

S3 File Breath data for Balbc control with chip. (PZFX)

S4 File Breath data of C57BL6 control with chip. (PZFX)

S5 File Breath data for Fig 2. Tumor Balbc animals.
(PZFX)

S6 File Breath data for Fig 5. CT26 tumor chipped mice.
(PZFX)

S7 File Breath data for Fig 5. B16F10 tumor chipped mice.
(PZFX)

S8 File Motion data for Fig 2. CT26 tumor Balbc.
(PZFX)

S9 File Motion data for Fig 3. Balbc control.
(PZFX)

S10 File Motion Balbc control with chip. (PZFX)

S11 File Motion C57BL6 control with chip. (PZFX)

S12 File Motion data for Fig 5. CT26 tumor chipped mice.
(PZFX)

S13 File Motion data for Fig 5. B16F10 tumor chipped mice.
(PZFX)
