# Supplementary material for: Automated monitoring of respiratory rate as a novel humane endpoint: A refinement in mouse metastatic lung cancer models
Source: PLoS One. 2021 Sep 20;16(9):e0257694. doi: 10.1371/journal.pone.0257694 (PMC8452061; doi:10.1371/journal.pone.0257694)
Supplement: S1 File — (DOCX) [file pone.0257694.s001.docx]

#

**Table A**. Comparison of increased breathing rates in CT26 and B16F10 tumor mice vs. those of control

| CT26 BALB/c | **CT26 tumor-bearing mice** | 25 | 26 | 27 | 30 | 31 | 33 |  |  | Mean ± SD |
| --- | --- | --- | --- | --- | --- | --- | --- | --- | --- | --- |
|  | **Breathing rate when motion speed began to decrease** | 230.6 | 243.6 | 217.0 | 245.0 | 305.5 | 236.9 |  |  | 246.4 ± 28.0*** |
|  | **Vehicle mice** | 15 | 16 | 17 | 22 | 23 | 24 |  |  |  |
|  | **Average breathing rate from days 23 to 29** | 168.6 | 175.2 | 158.7 | 160.1 | 158.1 | 160.2 |  |  | 163.5 ± 6.3 |
| CT26  BALB/c with UID chip | **CT26 tumor-bearing mice** | 22 | 27 | 28 | 33 | 34 | 36 | 38 | 40 | Mean ± SD |
|  | **Breathing rate when motion speed began to decrease** | 361.6 | 334.7 | 255.4 | 256.4 | 300.9 | 294.0 | 267.2 | 255.2 | 290.7 ± 37.6*** |
|  | **Vehicle mice** | 02 | 05 | 06 | 08 | 10 | 12 | 14 | 19 |  |
|  | **Average breathing rate from days 17-22** | 152.7 | 177.7 | 185.9 | 177.6 | 171.5 | 178.0 | 158.4 | 163.1 | 170.6 ± 10.7 |
| B16F10  C57BL/6 with UID chip | **B16F10 tumor-bearing mice** | 64 | 66 | 68 | 70 | 74 | 76 | 78 | 80 |  |
|  | **Breathing rate when motion speed began to decrease** | 220.8 | 208.7 | 216.7 | 322.0 | 229.2 | 320.6 | NA | 281.3 | 257.0 ± 46.1** |
|  | **Vehicle mice** | 44 | 46 | 48 | 50 | 52 | 54 | 56 | 58 |  |
|  | **Average breathing rate from days 20-28** | 197.0 | 193.3 | 219.3 | 201.3 | 200.8 | 182.7 | 203.4 | 202.7 | 200.1 ± 9.7 |

**: P<0.01; ***: P <0.001, unpaired student *t*-test

**Table B**. Pathology scores of animals without breathing rate increases

| **Experiment**  **(Animal strain)**  **(Tumor type)** | **Animal Number** | **Lung pathology score**  **(%)** | **Range of pathology scores** |
| --- | --- | --- | --- |
| CT26 BALB/c | 28 | 10 | 10 - 40 |
|  | 29 | 10 |  |
|  | 32 | 40 |  |
| CT26  BALB/c with UID chip | 24 | 10 | 10-20 |
|  | 30 | 20 |  |
| B16F10  C57BL/6 with UID chip | 62 | 0 | 0-30 |
|  | 72 | 30 |  |

**Table C**. Comparison of breathing rate in BALB/c mice with published data

| **Breathing rate (BPM)** | **Sex** | **Age**  **(wk)** | **Restraint** | **Measurement method** | **References** |
| --- | --- | --- | --- | --- | --- |
| 217 ± 6 | male | 7-11 | yes | transducer | 1 |
| 306 ± 5  303 ± 5 | male  female | 12-15 | yes | whole-body plethysmograph | 2 |
| 332 ± 24 | female | 9 | yes | whole-body plethysmograph | 3 |
| 423 ± 4 | male | 12 | no | whole-body plethysmograph | 4 |
| 358 ± 16 | male | 7 | no | whole-body plethysmograph | 5 |

**References:**

1. Muraki T, Kato R. Strain difference in the effects of morphine on the rectal temperature and respiratory rate in male mice. Psychopharmacology (Berl). 1986;89(1):60-4. doi: 10.1007/bf00175190. PubMed PMID: 3090593.

2. van Schaik SM, Enhorning G, Vargas I, Welliver RC. Respiratory syncytial virus affects pulmonary function in BALB/c mice. J Infect Dis. 1998;177(2):269-76. doi: 10.1086/514208. PubMed PMID: 9466511.

3. DeLorme MP, Moss OR. Pulmonary function assessment by whole-body plethysmography in restrained versus unrestrained mice. J Pharmacol Toxicol Methods. 2002;47(1):1-10. doi: 10.1016/s1056-8719(02)00191-0. PubMed PMID: 12387933.

4. Vanoirbeek JA, Rinaldi M, De Vooght V, Haenen S, Bobic S, Gayan-Ramirez G, et al. Noninvasive and invasive pulmonary function in mouse models of obstructive and restrictive respiratory diseases. Am J Respir Cell Mol Biol. 2010;42(1):96-104. doi: 10.1165/rcmb.2008-0487OC. PubMed PMID: 19346316.

5. Currie WD, van Schaik SM, Vargas I, Enhorning G. Ozone affects breathing and pulmonary surfactant function in mice. Toxicology. 1998;125(1):21-30. doi: 10.1016/s0300-483x(97)00158-3. PubMed PMID: 9585097

**Fig. A**





**Fig. A |** Breathing rate and motion speed in 8 vehicle injected BALB/c mice from day 13 to 23 in CT26 BALB/c with UID chip experiment. There were no changes in breathing rate or motion speed during the 14 days.

**Fig. B**





**Fig. B |** Comparison of body weight gain between CT26 tumor cell-injected animals (A for experiment without UID chip and B for experiment with UID chip) and corresponding vehicle injected animals as well as between B16F10 tumor-injected animals and corresponding control animals. There was no statistically significant difference between the vehicle and CT26 or B16F10 tumor animals (all P > 0.05).

**Fig. C**





**Fig. C |** Comparison of body temperatures taken in morning (A & C) or afternoon (B & D) differences between CT26 tumor cell- (A & B) or B16F10 cell-injected (C & D) animals and vehicle injected animals in all experiments. There was no statistically significant difference between the vehicle and CT26 or B16F10 tumor animals (all P > 0.05, mixed effects analysis).

**Fig D.**

**

**

**Fig. D** | Breathing rate and motion speed in 6 representative vehicle injected C57BL/6 mice from day 13 to 29. There were no changes in breathing rate or motion speed during the 14 days (both P > 0.05).
